# MEDI-SLATE: medical imaging slide-lecture aligned teaching ensemble

**DOI:** 10.1186/s42492-026-00218-0

**Published:** 2026-04-14

**Authors:** Motaleb Hossen Manik, Zabirul Islam, Ge Wang

**Affiliations:** 1 Department of Computer Science, Rensselaer Polytechnic Institute, Troy, NY 12180 United States; 2 Department of Biomedical Engineering, Rensselaer Polytechnic Institute, Troy, NY 12180 United States

**Keywords:** Medical imaging, Multimodal learning, Vision-language model, Educational dataset, Medical imaging education

## Abstract

Slide-based lectures remain the primary means by which undergraduate students learn about the mathematical, physical, and systems-level foundations of medical imaging. However, despite their central educational role, no openly available dataset pairs imaging lecture slides with clean, well-aligned explanatory narration suitable for scientific and educational research. The authors introduced MEDI-SLATE: medical imaging slide-lecture aligned teaching ensemble, constructed from a complete undergraduate biomedical engineering medical imaging course. The dataset contains 1117 high-resolution slides paired with refined narration derived from classroom audio through automatic speech recognition, followed by careful manual cleanup. MEDI-SLATE encompasses linear systems, Fourier analysis, signal processing, X-ray physics, computed tomography, positron emission tomography/single photon emission computed tomography, magnetic resonance imaging , ultrasound, and optical imaging. In addition to the slide-text pairs, the dataset includes lecture-level difficulty tags, key ideas, common student misunderstandings, and practice questions sourced directly from the instructor’s materials. A fully reproducible preprocessing pipeline covering slide extraction, narration refinement, alignment, and corpus-level analyses is provided. MEDI-SLATE offers a high-fidelity, openly available resource for medical imaging education, curriculum development, multimodal learning research, and creation of artificial intelligence-assisted instructional tools, with all data and codes released for transparent use and future extension.

## Introduction

Medical imaging is a core component of undergraduate biomedical engineering (BME) curricula that introduces students to mathematically intensive concepts spanning Fourier analysis, convolution, acquisition physics, system modeling, and modality-specific principles. In typical classroom settings, these ideas are communicated through slide-based lectures enriched by real-time instructor narration, which provides intuition, connects mathematical steps, and interprets diagrams and imaging geometries. Although such materials are essential for learning, no publicly accessible dataset currently pairs imaging lecture slides with refined, well-aligned explanatory texts suitable for multimodal educational research.

Existing lecture datasets generally emphasize broad, nontechnical subjects, short video snippets, or unprocessed recordings, rendering them poorly suited to the rigor of medical imaging coursework. Conversely, standard medical imaging datasets focus on clinical images and diagnostic labels rather than on the foundational principles taught in university classrooms. Researchers lack domain-specific, pedagogically coherent lecture materials that accurately reflect how imaging concepts are introduced to undergraduate students, thereby limiting progress in multimodal learning, scientific diagram understanding, and artificial intelligence (AI)-driven educational tools.

Another challenge stems from the multimodal structure of imaging lectures. Slides often contain equations, system diagrams, acquisition geometries, plots, and annotated images, whereas spoken narration provides context, emphasizes key steps, and disambiguates visual elements. Aligning narration with slide content requires handling automatic speech recognition (ASR) artifacts, terminology inconsistencies, and temporal drift in how instructors reference diagrams or mathematical expressions. These characteristics render slide-text alignment both pedagogically valuable and technically nontrivial.

To address these requirements, we introduced MEDI-SLATE, a curated multimodal dataset developed from a complete undergraduate medical imaging course in BME. The dataset comprises 1117 high-resolution slide images paired with refined narration derived from classroom audio through ASR, followed by meticulous manual correction. The course includes foundational content–linear systems, Fourier methods, and signal processing–and modality-focused lectures on X-ray imaging, computed tomography (CT), positron emission tomography/single photon emission computed tomography (PET/SPECT), magnetic resonance imaging (MRI), ultrasound, and optical imaging. The resulting materials provide a comprehensive and coherent representation of the undergraduate imaging curriculum.

Beyond the aligned slide-text pairs, MEDI-SLATE incorporates additional educational aids drawn directly from the instructor’s materials, including difficulty levels, key conceptual insights, common misunderstandings, and practice questions. These enrichments strengthen the dataset’s utility for curriculum analysis, instructional design, and the development of adaptive learning tools. A unified preprocessing pipeline was released alongside the dataset, enabling slide extraction, narration refinement, alignment, and reproducible corpus-level statistical analyses.

Overall, these components position MEDI-SLATE as a high-fidelity resource for medical imaging education and a foundation for research on multimodal lecture understanding, scientific vision-language reasoning, and educational AI. The remainder of this paper details dataset construction, preprocessing workflow, corpus statistics, educational enhancements, and potential directions enabled by this release.

To put MEDI-SLATE in perspective, we briefly review the existing efforts in medical imaging education, multimodal lecture resources, scientific diagram reasoning, and educational natural language processing (NLP). Previous studies have explored how imaging concepts are taught, developed datasets for slide- or document-based understanding, and introduced multimodal scientific QA benchmarks. However, none of these efforts provides a coherent, domain-specific collection of high-fidelity slides paired with a refined, instructor-style narration spanning the entire medical imaging curriculum. This subsection summarizes these areas and highlights the gaps addressed by MEDI-SLATE.

Prior studies have examined how imaging is taught and the challenges that arise in communicating mathematically intensive, physics-driven concepts. Grover et al. [1] provided a clinician-oriented introduction to MRI, emphasizing the difficulties that students encounter when learning complex imaging physics without a structured visual explanation. Altun et al. [2] surveyed modern radiology teaching methods, including flipped classrooms and digital platforms, and highlighted the importance of multimodal technology-enabled instruction. Harthoorn et al. [3] identified gaps in radiology education, highlighting inconsistent physics coverage and the limited availability of high-quality instructional materials. Wade et al. [4] synthesized evidence for effective radiology teaching strategies and found that structured visual explanations and well-sequenced content significantly improve the learning outcomes.

Collectively, these studies demonstrate the need for accessible, consistent, multimodal educational resources. MEDI-SLATE provides the first publicly available slide-narration dataset, capturing a full medical imaging curriculum with refined, reliable explanations.

Although several datasets explore visually rich educational materials, none provides narration-aligned scientific slides. SlideVQA [5] focuses on visual question answering over multislide inputs, but lacks explanatory narration or full-lecture continuity. The MLP dataset [6] includes slides and transcripts from broad, nontechnical presentations; however, its textual content has not been refined for scientific correctness. LecSlides-370K [7] scales to hundreds of thousands of slide decks across many disciplines, yet does not include expert narration, domain-specific refinement, or a coherent course-level structure.

Compared to these datasets, MEDI-SLATE uniquely provides domain-specific slide images paired with refined narration, enabling research that requires both visual and explanatory accuracy within a coherent imaging curriculum.

Work in scientific QA targets diagrams, graphs, and structured reasoning; however, these efforts focus on isolated questions rather than on sustained instructional content. Learn-to-explain [8] introduces multimodal chain-of-thought reasoning for science QA, demonstrating the importance of explicit reasoning steps grounded in diagrams. SciGraphQA [9] provides QA pairs on synthetic scientific graphs, emphasizing numerical and visual interpretations. SPIQA [10] evaluates multimodal reasoning in scientific papers, including figures, tables, and diagrams.

Although valuable for diagram understanding, these datasets do not include slide decks, provide instructor-style narration, or reflect course-level pedagogical structures. MEDI-SLATE captures the full instructional context of scientific diagrams, equations, and imaging schematics within university courses.

Other lines of work have focused on textual educational resources and the generation of presentations. LectureBank [11] models prerequisite relationships among NLP lectures, showing the value of structured educational corpora, but remains text-only. DOC2PPT [12] generates presentation slides from scientific documents, thereby highlighting the role of document semantics in educational designs. MEDI-SLATE provides a complementary direction: understanding existing scientific slides aligned with expert narrations.

Recent vision-language models (VLM) such as LLaVA [13], BLIP-2 [14], and SOLO [15] demonstrate strong multimodal capabilities; however, their training relies heavily on natural images, web diagrams, or general-purpose captions. These models have limited exposure to domain-specific scientific slides, mathematical derivations, or modality physics.

By supplying a refined narration aligned with high-fidelity scientific slides, MEDI-SLATE can serve as a benchmark for evaluating and improving scientific VLMs in domains that require equation interpretation, geometric reasoning, and physics-aware understanding.

In summary, existing work demonstrates a growing interest in multimodal educational resources, scientific diagram understanding, and lecture-centric NLP. However, no prior dataset provides high-fidelity scientific slides paired with refined, instructor-style narration across a coherent undergraduate medical imaging curriculum. MEDI-SLATE addresses this gap by offering a domain-specific, pedagogically grounded multimodal resource that supports both educational research and scientific vision-language modeling.

## Methods

This section includes the dataset description along with the methodological workflow.

### Dataset description

MEDI-SLATE is a multimodal educational dataset containing 1117 aligned slide-text pairs derived from a complete undergraduate BME medical imaging course. Each slide is paired with a refined narration segment to enable multimodal learning, retrieval, and analysis of scientific language based on visual instructional content. The course covers foundational concepts central to undergraduate imaging education, including mathematics, signal processing, physics, algorithms, instrumentation, and modality-specific principles, such as radiography, CT, PET/SPECT, MRI, ultrasound, and optical imaging.

#### Lectures and curriculum scope

**Fig. 1:**
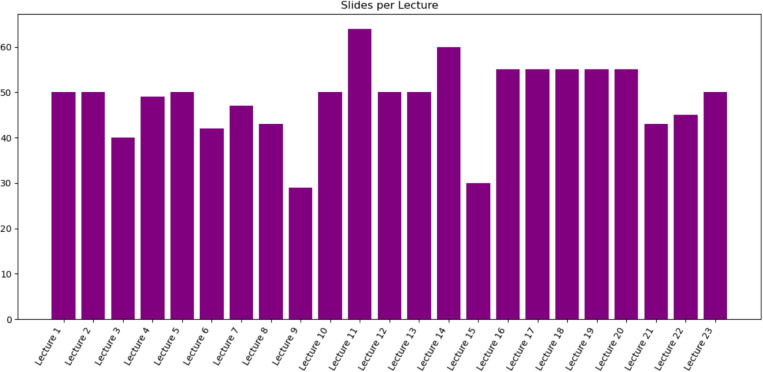
Number of slides per lecture in MEDI-SLATE. Each bar corresponds to one of the 23 lectures

The dataset consists of 23 full lectures corresponding to a semester-long introductory course in medical imaging. Individual lectures contain 29–64 slides, reflecting natural variations in topic difficulty and instructional emphasis. Figure 1 shows the distribution of slide counts across the curriculum.

The course content spanned the following:

- Mathematical foundations: linear systems, convolution, Fourier analysis, and signal processing;
- Modality principles: radiation emission and attenuation, magnetic resonance and relaxation, wave propagation and interference, imaging geometry, collimation, k-sampling, ultrasound beamforming, and optical interferometry;
- Instrumentation: sources, detectors, collimators, magnets, coils, and transducers;
- Image quality: resolution, noise, artifacts, system response, and performance metrics.

This scope reflects the structure of typical undergraduate imaging curricula and offers diverse visual and textual materials that are well-suited to BME education and multimodal scientific reasoning.

#### Slide images

The slide images were exported directly from the instructor’s original presentation files as high-resolution joint photographic experts groups (JPEGs). To maximize visual fidelity, we used a PDF intermediate workflow–exporting slides from PowerPoint to PDF, then to JPEG at maximum quality settings–preserving vector graphics, mathematical equations, and fine diagram details. These slides contain the following:

- Mathematical formulas and stepwise derivations,
- Annotated diagrams and physical schematics,
- Modality-specific examples such as projections and reconstructions,
- Plots, graphs, and signal-processing visualizations.

**Fig. 2:**
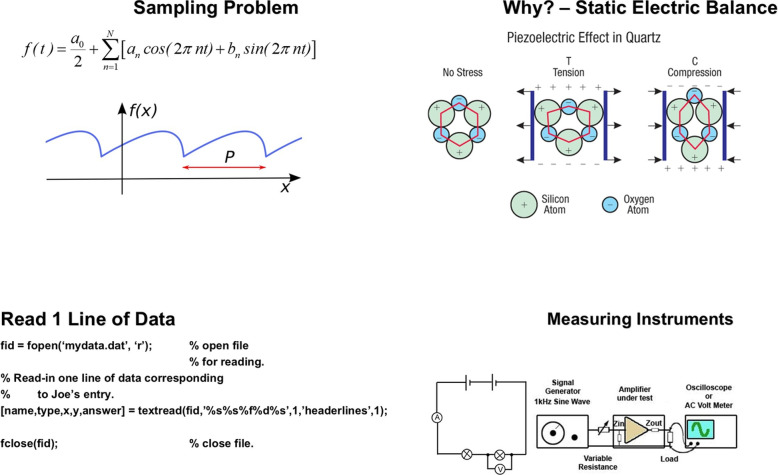
Representative gallery of slides from MEDI-SLATE, showing mathematical derivations, physical illustrations, modality examples, and scientific plots typical of undergraduate imaging instruction

Compared to natural image datasets, these slides contain structured scientific diagrams, mathematical notations, and dense conceptual content, offering a visually rich yet technically demanding setting for multimodal representation learning. A representative gallery is presented in Fig. 2.

#### Refined narration text

For each slide, the corresponding narration was extracted from classroom audio recordings. Whisper ASR was used to produce an initial transcript, followed by a multistage refinement process as follows:

- Remove filler words and disfluencies,
- Correct specialized terminology (e.g., *k*-space, attenuation, backprojection),
- Standardize phrasing for clarity,
- Preserve the instructor’s pedagogical intent.

During refinement, occasional temporal drifts between ASR timestamps and actual slide transitions required manual correction, particularly when the instructor referenced diagrams spanning multiple slides. These adjustments ensured that each narration segment corresponded accurately to its visual counterpart, yielding clean and coherent slide-text pairs suitable for multimodal analysis.

#### Dataset structure

MEDI-SLATE has a consistent directory structure that supports reproducibility and integration with downstream pipelines. Each lecture folder contains the Images/, Texts/, and Final/ directories. The first two contain extracted slide images and narration files, whereas the Final/ directory provides synchronized slide-text pairs ready for multimodal research.
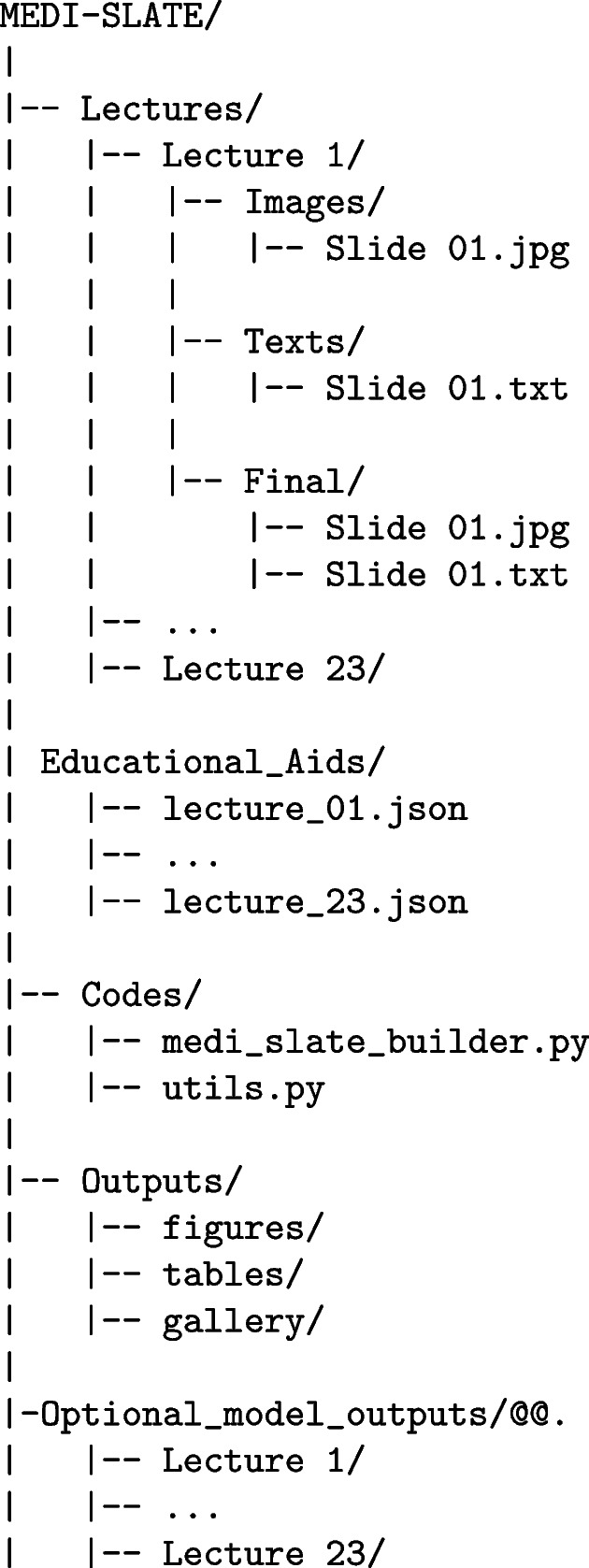


This directory scheme ensures clear indexing (Lecture X/Slide Y) and separation of raw data, processed data, educational aids, and optional model outputs.

#### Corpus summary

Corpus-level statistics are summarized in Table 1, including total slide count, narration token count, and vocabulary size. The per-lecture statistics are presented in Table 2. Overall, these summaries highlight the combination of conceptual slides, geometric diagrams, derivation-heavy content, and explanatory text characteristics of undergraduate imaging lectures.

**Table 1:** Global summary of MEDI-SLATE, including total slide number, token count, and vocabulary size

Total lecture	Total slide	Total token	Average slides per lecture	Average tokens per lecture
23	1117	262182	48.565217	11399.217391

**Table 2:** Per-lecture statistics on slides, narration tokens, and vocabulary across the 23 undergraduate medical imaging lectures

Lecture	lecture_num	num_slides	num_tokens	vocab_size
Lecture 1	1	50	9294	2722
Lecture 2	2	50	5329	1671
Lecture 3	3	40	3533	1374
Lecture 4	4	49	9000	1951
Lecture 5	5	50	19816	2824
Lecture 6	6	42	12758	2199
Lecture 7	7	47	15722	2422
Lecture 8	8	43	10128	1873
Lecture 9	9	29	5788	1408
Lecture 10	10	50	10321	2510
Lecture 11	11	64	14633	3033
Lecture 12	12	50	11397	2755
Lecture 13	13	50	9134	1990
Lecture 14	14	60	9362	2661
Lecture 15	15	30	5271	1468
Lecture 16	16	55	11694	2802
Lecture 17	17	55	10834	2702
Lecture 18	18	55	10835	2241
Lecture 19	19	55	15023	2774
Lecture 20	20	55	16805	3575
Lecture 21	21	43	15750	3114
Lecture 22	22	45	11224	2560
Lecture 23	23	50	18531	3614

### Methodological workflow

**Fig. 3:**
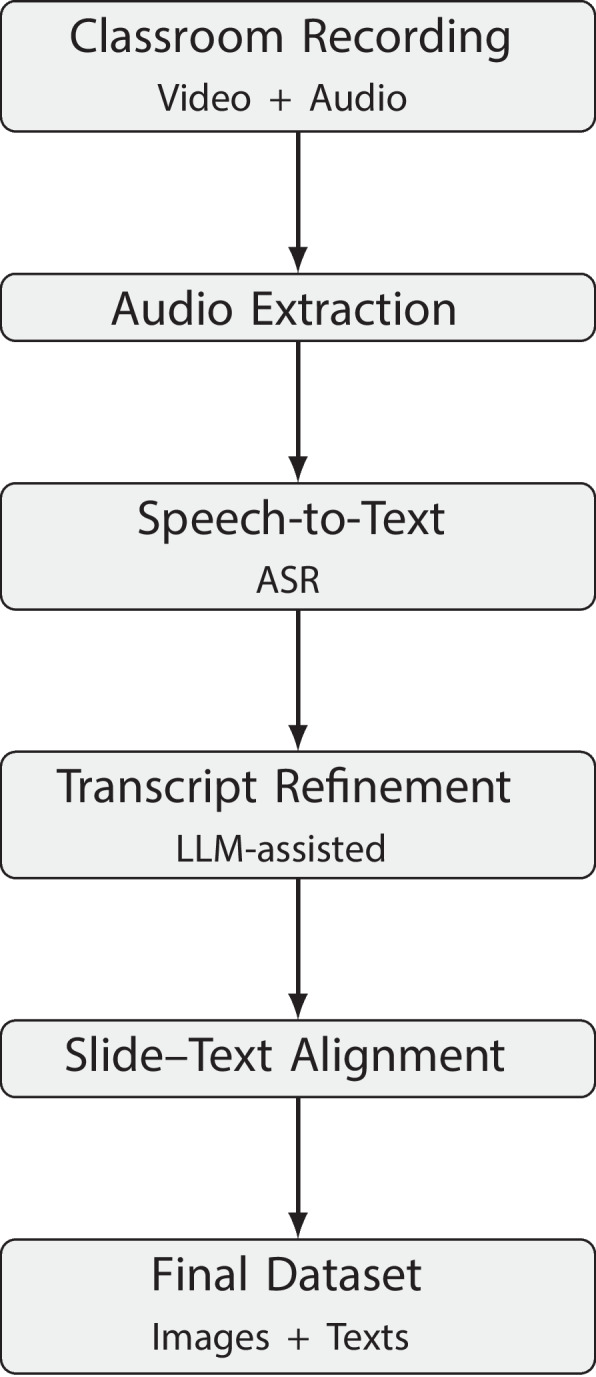
End-to-end processing workflow transforming lecture recordings and presentation slides into aligned slide-text pairs

To enable consistent and reproducible construction of MEDI-SLATE, we developed a unified end-to-end methodological workflow that transforms raw instructional materials into fully aligned slide-text pairs, structured metadata, and corpus-level statistics. The major methodological stages are summarized in Fig. 3.

#### Slide export

The slides were directly exported from the instructor’s original presentation files as high-resolution JPEG images. This preserved the mathematical notation, diagram fidelity, and visual clarity of the scientific figures. A consistent naming scheme (Slide_XX.jpg) ensured deterministic mapping between the slides and their paired narration segments.

#### ASR transcription

The classroom audio was transcribed using Whisper ASR. During pipeline development, we compared three Whisper variants (base, medium, and large-v3) on a representative subset of lecture audio to select a practical configuration for long-form classroom speech containing technical terminology. Based on a qualitative inspection of the computational efficiency, transcript clarity, and terminology recognition, we selected the medium model. It provided sufficiently accurate transcripts for downstream refinement and slide alignment while requiring substantially less computational cost than large-v3 for processing the full 23-lecture dataset. This model was chosen because of its robustness to

- Technical vocabulary common in imaging physics,
- Variable speaking pace during live teaching,
- Moderate ambient classroom noise,
- Long-form explanations spanning multiple sentences.

Whisper provided first-pass transcripts with approximate sentence boundaries and timing estimates.

#### Transcript refinement

Raw ASR transcripts contain fillers, fragmented phrases, and occasional terminological errors. A controlled refinement stage was applied to obtain a clean, readable narration while faithfully preserving the instructional content.

##### Model specification

Transcript refinement was performed using GPT 5.1 (OpenAI API) software. The model was prompted to perform the following:

- Correct terminology (e.g., “kspace” $\rightarrow$ “k-space”),
- Remove disfluencies and repetitions,
- Restore complete sentences and coherence,
- Maintain the instructor’s original explanatory meaning.

Two PhD-level annotators manually reviewed all refined transcripts for fidelity to the original audio. Occasional phrasing drift was corrected to ensure that the final text aligned with the instructor’s spoken content.

#### Slide-text alignment

A one-to-one slide-text alignment was produced by combining

- ASR timestamps indicating when each slide was discussed,
- Slide-change cues observed in the lecture videos,
- Manual review to verify correctness.

##### Alignment challenges

Temporal drift occasionally occurred between ASR timestamps and visual slide transitions–typically during rapid explanations, multislide derivations, or when the instructor referenced diagrams that spanned several slides. Approximately 8%–12% of the slide boundaries required manual correction. Ambiguities were resolved by inspecting the lecture video timeline and matching verbal cues (e.g., “as shown here”) to the corresponding visual elements. In practice, manual alignment effort scaled with slide transition frequency: for dense lectures with frequent slide changes, alignment required approximately half the lecture duration (e.g., $\sim$40 minutes for an 80-minute lecture), whereas lectures with fewer transitions typically required about one-third (approximate wall-clock time). This step was performed by a single PhD-level annotator using a simple pause/resume workflow. These adjustments ensured accurate high-fidelity slide-text pairing for multimodal learning tasks.

#### Generation of educational aids

MEDI-SLATE includes lecture-level educational aids–difficulty tags, key ideas, common misunderstandings, and multiple-choice questions (MCQs)–created manually by the authors. In contrast to transcript cleanup, which primarily includes linguistic normalization, educational aid creation requires pedagogical abstraction and error-sensitive conceptual structuring. Although large language model-assisted generation was considered, the authors’ complete expertise with the course content rendered manual creation more efficient and accurate than automated approaches requiring extensive verification. All items were strictly derived from the slide content and refined narration to ensure alignment with the instructor’s pedagogical intent. Materials focused exclusively on imaging physics, mathematical reasoning, and modality principles without introducing additional or speculative content.

The finalized aids are stored in Educational_Aids/ as JavaScript object notation (JSON) files (lecture_XX.json), each containing the following:

- MCQs linked to specific conceptual points,
- Difficulty-level annotations,
- Common misunderstandings observed among learners.

These educational aids distinguish MEDI-SLATE from previous slide datasets by directly supporting curriculum analysis and instructional design.

##### Annotation protocol

Two PhD-level annotators independently proposed annotations, with a final review by a course instructor. Disagreements were resolved through discussions. For a subset of 50 annotated items, annotators achieved a Cohen’s $\kappa$ of 0.75 for difficulty labels, indicating substantial agreement.

#### Corpus statistics and figure generation

The script medi_slate_builder.py automatically computes the following:

- Per-slide and per-lecture sentence, token, and vocabulary statistics;
- Global corpus vocabulary distributions;
- Dataset-wide visualizations (histograms, word clouds, and slide gallery);
- LaTeX-ready tables for summary statistics.

All the figures used in this paper (Fig. 1) were generated directly using this pipeline to ensure complete reproducibility.

#### Final assembly and packaging

The final dataset release included the following:

- Slide images and refined narration text;
- Structured metadata in CSV/JSON formats;
- Educational aids for all lectures;
- Corpus summaries, figures, and LaTeX tables;
- Optional model outputs (auxiliary material);
- All processing scripts necessary for complete reconstruction.

This design supports transparent dataset construction and facilitates future extensions, including additional courses, enhanced multimedia alignment, and fine-grained diagram annotation.

## Results

We conducted a comprehensive quantitative analysis of all 1117 slide-text pairs in MEDI-SLATE to characterize the linguistic structure, narration density, topic coverage, and scientific terminology usage. The goal of this analysis was to document how undergraduate-level medical imaging concepts are conveyed through various combinations of mathematical derivations, diagrams, and spoken explanations.

### Sentence distribution per slide

**Fig. 4:**
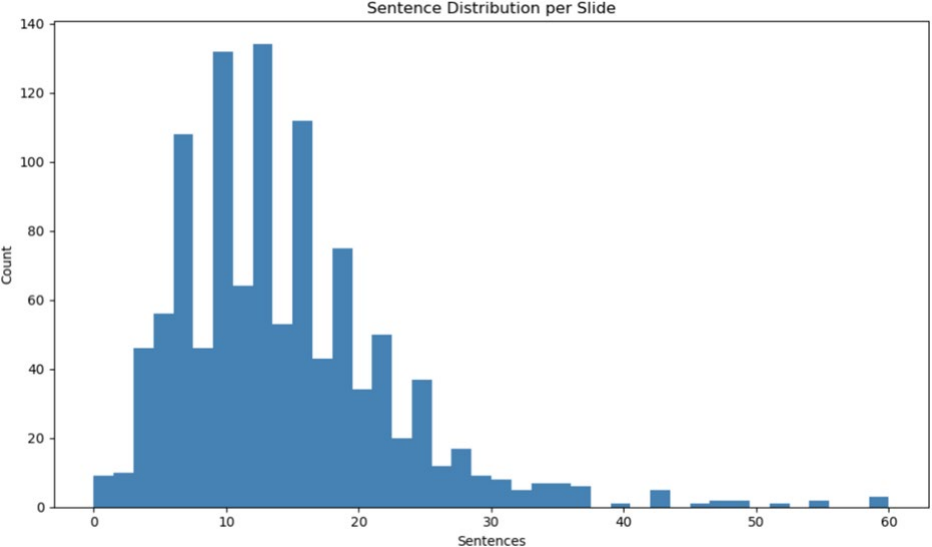
Distribution of sentence counts per slide across MEDI-SLATE. Although many slides involve concise narration, mathematically detailed slides require substantially longer verbal explanations

The slides exhibited substantial variability in the length of their associated narrations. Figure 4 shows the distribution of the sentence counts per slide. Most slides include 8–20 sentences, reflecting concise conceptual summaries or short derivations. By contrast, slides that introduce multistep mathematical developments or detailed system descriptions contain significantly longer narration segments.

This spread reflects authentic instructional pacing: some slides anchor a concept in a few sentences, whereas others support extended derivations or multiconcept discussions.

### Token distribution per slide

**Fig. 5:**
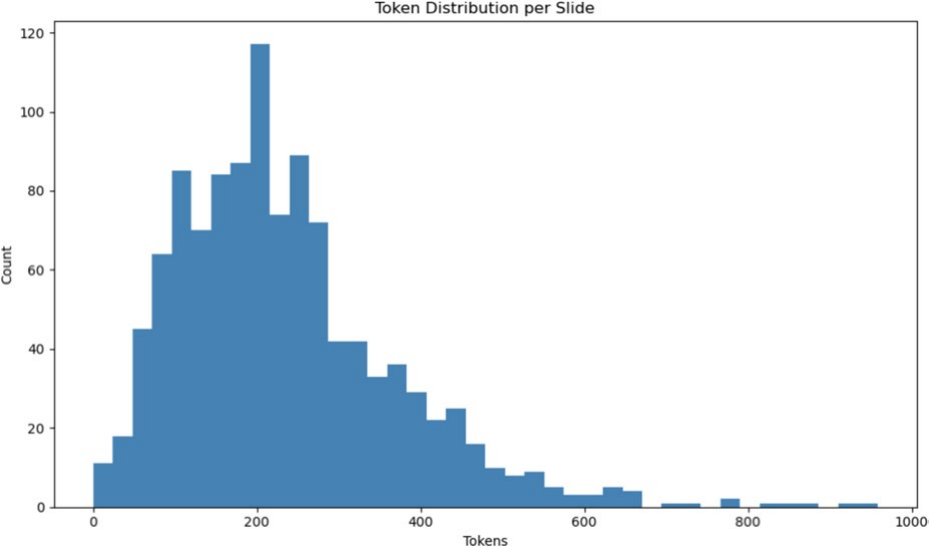
Token distribution across all slides. Most slides fall within a moderate range, whereas mathematically intensive slides contain significantly longer narration

Token-level statistics provide a complementary view of narration density. Figure 5 shows the distribution of the total tokens per slide. The majority fall within the range of 120–300 tokens; however, some exceed 900 tokens, particularly those containing multistep derivations, system-level workflows, or complex imaging geometries.

These variations create a realistic spectrum of input lengths for models trained with scientific and educational content.

### Token distribution per lecture

**Fig. 6:**
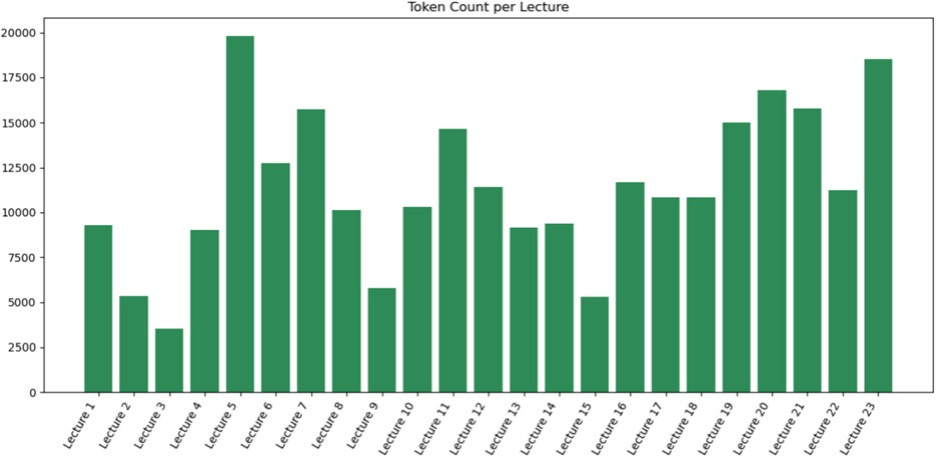
Total token count per lecture. Mathematical and signal-processing topics require longer narration, whereas modality introductions exhibit shorter segments

Narration length varies across lectures depending on the topic. Figure 6 illustrates the total number of narration tokens per lecture. Lectures covering mathematical foundations, such as Fourier analysis, reconstruction, and linear systems theory, have substantially higher token counts than modality overview lectures, which rely substantially on diagrams and qualitative explanations.

This pattern aligns with common undergraduate imaging curricula, where mathematically intensive topics demand deeper instructor explanations.

### Domain terminology and topic distribution

**Fig. 7:**
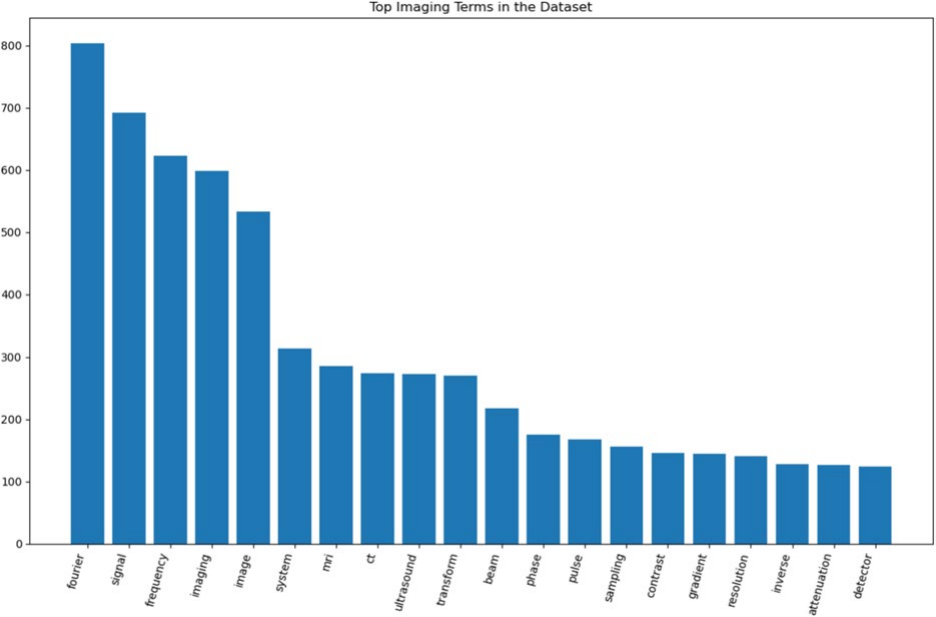
Most frequent imaging-related terms extracted from narration text. Vocabulary spans mathematics, imaging physics, reconstruction, and modality-specific terminology

To assess the scientific vocabulary coverage, we extracted the most frequent imaging-related terms from the full corpus. Figure 7 shows the key terms spanning imaging physics (e.g., attenuation, gradient, and frequency), modality mechanics (e.g., projection, beamforming, and k-space), and reconstruction principles (e.g., sampling and transform).

This distribution confirms that narration preserves the scientific depth of the course.

### Word cloud visualization

**Fig. 8:**
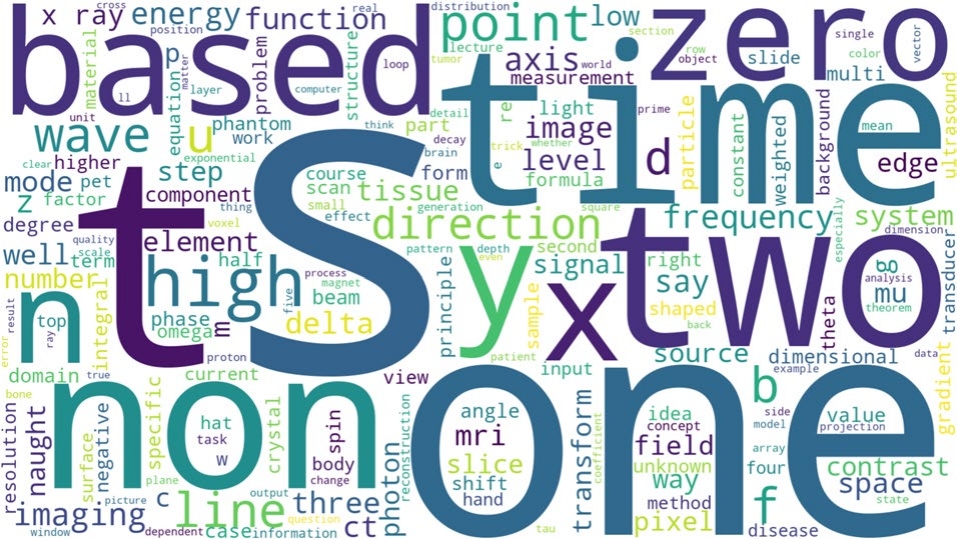
Word cloud generated from the global vocabulary distribution. Prominent terms correspond to mathematical methods, imaging physics, and modality concepts

Figure 8 shows the overall vocabulary distribution. The dominant terms emphasize the mathematical and physical foundations of the course, demonstrating that the refined narration retains instructional accuracy and domain specificity.

### Summary of findings

The quantitative analysis of MEDI-SLATE highlights the following key characteristics:

- Authentic instructional pacing: Narration lengths vary across slides and lectures, reflecting real classroom delivery.
- Rich interdisciplinary vocabulary: The corpus integrates terminology from mathematics, physics, imaging systems, algorithms, and instrumentation.
- Diverse scientific visual content: Slides include equations, diagrams, system models, plots, and modality-specific illustrations.
- Topic-dependent variation: Mathematical lectures contain significantly more narration than modality overview lectures, reflecting typical curricular structure.
- Educational suitability: The corpus structure supports multimodal alignment, representation learning, and educational AI tasks based on scientific content.

Overall, these statistics show that MEDI-SLATE captures the complexity and depth of undergraduate medical imaging instruction and provides a scientifically validated multimodal resource for research in educational systems and domain-specific language modeling.

## Discussion

MEDI-SLATE introduced the first multimodal dataset that pairs high-resolution lecture slides with refined instructional narration spanning a complete undergraduate medical imaging course. The dataset captures the mathematical, physical, and conceptual foundations of imaging science, providing a structured representation of topics such as X-ray physics, CT geometry, MRI k-space acquisition, ultrasound beamforming, PET/SPECT principles, and multidimensional signal processing. This breadth reflects authentic instructional practices and makes MEDI-SLATE a valuable resource for research on multimodal learning, scientific reasoning, and educational technologies.

The multimodal nature of the dataset enables several research directions. Slide-text pairs support lecture understanding, cross-modal retrieval, multimodal summarization, and domain-specific representation learning. The slides feature dense equations, imaging geometries, and scientific diagrams, whereas the narration provides a structured explanation and mathematical interpretation. These characteristics expose challenges for current general-purpose VLMs, which are typically trained on natural images and web-derived captions rather than technical educational materials. Consequently, MEDI-SLATE offers a demanding and realistic setting for developing imaging- and science-aware multimodal models. Despite its strengths, this dataset has several limitations as follows.

### Limitations

#### Educational content only

The dataset contains educational slides and narration, but not clinical images or diagnostic materials. Therefore, it should not be used for clinical predictions or patient-facing applications.

#### Slide-level alignment

The narration aligned with the level of the entire slide. The dataset does not specify region-level grounding between text spans and diagram components such as equations, figures, or axes.

#### Residual ASR artifacts

Although the narration has been refined for clarity, minor transcriptional inaccuracies may remain, particularly on slides with dense equations or rapid explanations.

#### Single-course scope

The dataset reflects a single undergraduate course taught by an instructor. Although this ensures consistency, it also limits stylistic and curricular diversity. Future releases should incorporate additional courses by various instructors and institutions.

#### Complex scientific content

The slides contain specialized diagrams, mathematical derivations, and physics-based schematics that pose significant challenges for current VLMs.

### Ethical considerations

MEDI-SLATE was created with careful attention paid to educational integrity and ethical data handling.

#### No patient data

The dataset contains no patient information, clinical images, or radiological case material and therefore poses no privacy risk.

#### Instructor permission

All instructional materials were created by a course instructor who provided explicit permission for their public release.

#### Responsible use

The dataset is intended for use in educational AI, multimodal learning, and scientific reasoning research. It should not be used for diagnostic, clinical, or patient-facing applications.

### Applications

MEDI-SLATE supports a broad set of research directions at the intersection of multimodal learning, educational AI, scientific diagram understanding, and domain-specific language modeling. As the dataset contains precisely aligned slide images and refined instructional narration from an entire undergraduate medical imaging course, it provides a realistic platform for jointly interpreting visual, mathematical, and conceptual information.

#### Multimodal lecture understanding

The one-to-one alignment between the slides and narration enables tasks such as slide-to-text retrieval, text-to-slide indexing, lecture summarization, and multimodal representation learning. These tasks are central to building systems that can navigate and organize long-form instructional materials, supporting applications for lecture indexing and automated course construction.

#### Educational AI and tutoring systems

Refined narration enables curriculum-aware educational AI systems to answer questions, clarify concepts, generate practice questions, and provide adaptive tutoring. Because the text is clean, coherent, and instructional–rather than a raw ASR output–models trained on MEDI-SLATE can more reliably explain the course structure and pedagogical intent.

#### Scientific vision-language reasoning

Many slides contain equations, multistep imaging geometries, annotated diagrams, and signal-processing plots. These features render MEDI-SLATE a valuable resource for developing models that connect mathematical expressions with verbal explanations, interpret scientific figures, and reason across physics-based visual content. The dataset bridges a gap in existing multimodal resources, which rarely include technical diagrams paired with expert narrations.

#### Domain adaptation and pretraining

Narration text provides a domain-specific corpus suitable for pretraining or adapting language models to medical imaging terminology. Potential applications include imaging-aware language modeling, curriculum-based representation learning, modality-specific fine-tuning for CT/MRI/ultrasound/nuclear medicine tasks, and transfer learning for educational or scientific NLP problems.

#### Future extensions

MEDI-SLATE is designed to serve as a foundation for future work. Planned extensions include fine-grained diagram grounding, integration of video-slide-text alignment, region-level annotation of equations and figures, and the inclusion of additional imaging courses taught by different instructors and institutions. These additions support broader generalizations and enable more challenging scientific reasoning.

In summary, MEDI-SLATE represents an important initial step toward building comprehensive multimodal educational resources for medical imaging. This establishes a structured and reproducible foundation on which future curriculum-scale datasets and scientific multimodal benchmarks can be developed.

## Conclusions

This study introduced MEDI-SLATE, a high-fidelity multimodal dataset comprising 1117 aligned slide-text pairs drawn from a full undergraduate medical imaging course. By combining refined instructional narration with domain-specific scientific slides, the dataset bridges a critical gap in multimodal educational research and provides a realistic testbed for imaging-aware machine learning. MEDI-SLATE has been released along with all the preprocessing scripts, metadata, and educational aids to ensure full reproducibility and extensibility. We envision that this dataset will serve as a foundation for future advances in multimodal scientific reasoning, lecture understanding, educational AI, and imaging-focused vision-language modeling, and anticipate that subsequent versions will expand curricular coverage and introduce additional annotation layers.

## Data Availability

All the data are available here: https://github.com/manikm-114/MEDI-SLATE.

## Appendix

This appendix provides metadata documentation, extended tables, and reproducibility notes that supplement the main text.

### 1 Metadata schema

MEDI-SLATE provides corpus-level and lecture-level statistics summarizing the dataset size and narration complexity. In the current release, these metadata are distributed as two LaTeX-ready tables (used directly in the main manuscript and also available in Github directory: MEDI-SLATE/outputs/tables): (1) a global summary table and (2) a per-lecture summary table. These tables were computed from the refined narration text files by counting tokens and unique vocabulary terms within each lecture.

#### Per-lecture statistics

The per-lecture table reports, for each lecture: the lecture index, number of slides, total narration tokens, and vocabulary size (unique tokens). This table is included in the manuscript source as table_per_lecture.tex and is used in Table 2.

#### Global summary statistics

The global table reports the total number of lectures, total number of slides, total narration tokens, and averages per lecture. This table is included in the manuscript source as table_summary.tex and is used in Table 1.

#### Reproducibility note

All values in these two tables are derived deterministically from the released slide-level narration text files (one .txt per slide). Specifically, token counts are computed by tokenizing the refined narration, and vocabulary size is computed as the number of unique tokens aggregated within each lecture. As a result, the lecture-level and corpus-level statistics can be regenerated from the dataset release without relying on any additional hidden metadata files.

### 2 Processing script and reproducibility

All dataset statistics, figures, and LaTeX tables in this paper can be regenerated using the unified builder script: python medi_slate_builder.py.

The script loads all slide images and narration text, computes per-slide and per-lecture linguistic statistics, extracts vocabulary and domain terminology, generates the distribution figures, and exports LaTeX-ready summary tables. The pipeline logs its own execution to ensure full reproducibility and facilitate community extensions.

### 3 Optional model outputs

As auxiliary material, we include automatically generated outputs from four vision-language models. These predictions were produced only to support qualitative error analysis and exploration of model behavior on scientific slides; they are not curated ground-truth annotations and should not be used as training labels. The outputs often contain hallucinations or scientific inaccuracies, reflecting the difficulty current models face when interpreting equations, imaging schematics, and physics-based diagrams.

These predictions can help researchers examine common failure modes, compare model behaviors on technical visual content, and study the challenges of scientific multimodal reasoning. All optional outputs are included in the public dataset release at: https://github.com/manikm-114/MEDI-SLATE.

They are organized by lecture in: Optional_model_outputs/.

This appendix concludes the supplementary documentation for the MEDI-SLATE dataset.

## Abbreviations

AI: Artificial intelligence
ASR: Automatic speech recognition
BME: Biomedical engineering
CT: Computed tomography
JPEG: Joint photographic experts group
JSON: JavaScript object notation
MCQ: Multiple-choice question
MRI: Magnetic resonance imaging
NLP: Natural language processing
PET: Positron emission tomography
SPECT: Single photon emission computed tomography
VLM: Vision-language model
